# Network Pharmacology and Experimental Validation-based Investigation of the Underlying Mechanism of Yi-Yi-Fu-Zi-Bai-Jiang-San of Nasopharyngeal Carcinoma

**DOI:** 10.7150/jca.109758

**Published:** 2025-03-29

**Authors:** Zehua lin, Ting Huang, Baoai Han, Zezhang Tao, Xiong Chen

**Affiliations:** 1 Department of Otolaryngology, Head and Neck Surgery, Zhongnan Hospital of Wuhan University, Wuhan, 430071, Hubei, China.; 2 Department of Otolaryngology, Head and Neck Surgery, Renmin Hospital of Wuhan University, Wuhan, 430071, Hubei, China.; 3 Sleep Medicine Centre, Zhongnan Hospital of Wuhan University, Wuhan, 430071, Hubei, China.

**Keywords:** Nasopharyngeal Carcinoma, Yi-Yi-Fu-Zi-Bai-Jiang-San, Acacetin, PTGS2

## Abstract

Yi-Yi-Fu-Zi-Bai-Jiang-San (YYFZBJS) is a representative traditional Chinese medicine (TCM) formula. However, its potential anti-tumor effects in nasopharyngeal carcinoma (NPC) remains unclear. This study aims to investigate the monomers of YYFZBJS and their associated targets in the treatment of NPC. The primary active compounds of YYFZBJS and their corresponding targets were identified using the TCMSP, SEA, and Super-PRED databases. NPC-related target proteins were retrieved from OMIM, GeneCards, and TTD databases. A protein-protein interaction network was constructed using the common target proteins of YYFZBJS active compounds and NPC. Core genes were identified through three algorithms in CentiScape 2.2. Gene Ontology (GO) and Kyoto Encyclopedia of Genes and Genomes (KEGG) analyses were then performed on these core genes. Validation was conducted using the GSE53819 and GSE13597 datasets. Finally, interactions between core targets and active ingredients were confirmed through molecular docking, molecular dynamics simulations, and cell-based experiments. A total of 715 corresponding to YYFZBJS active compounds and 3159 NPC-related targets were screened. Among these, 143 intersection genes were identified, from which 32 core genes were selected based on degree centrality, closeness centrality, and betweenness centrality. GO and KEGG analyses of these core genes revealed relevant biological processes and pathways. Furthermore, these 32 core genes were cross-referenced with the GSE53819 and GSE13597 datasets, identifying PTGS2 and CCND1 as valid targets of active compounds. Molecular docking, molecular dynamics simulations and cell experiments confirmed the effectiveness of the Acacetin-PTGS2 pathway. Acacetin of the main active ingredient in YYFZBJS suppressed NPC by downregulating PTGS2 expression.

## Introduction

Nasopharyngeal carcinoma (NPC) is a solid malignant tumor that originates in the nasopharynx. According to the International Agency for Research on Cancer, 120,416 new cases of NPC were reported in 2022, along with 73,476 deaths, ranking it 23rd among newly diagnosed cancers and 21st among cancer-related deaths worldwide 1. The etiology of NPC is multifactorial, with genetic susceptibility, environmental factors, and Epstein-Barr virus infection as significant contributors to its development 2. Despite the fact that chemoradiotherapy has emerged as the primary treatment for NPC, the 5-year survival rate for NPC patients remains low, with local recurrence and distant metastasis continuing to challenge effective management. Thus, identifying novel treatment strategies and adjuvant therapies is critical for improving patient survival rates and their quality of life.

In comparison to conventional chemotherapy, traditional Chinese medicine (TCM) has gained increasing attention due to its ability to regulate systemic functions, target multiple pathways, and produce fewer side effects. As research into herbal medicine expand, there is growing interest in exploring herbal remedies as potential agents for tumor treatment. Coicis Semen, Aconiti Lateralis Radix Praeparata, and Herba Patriniae are notable Chinese herbal formulas. Coicis Semen is commonly used in TCM for clearing heat and dampness, promoting drainage, and exhibiting potential anti-tumor effects 3. Aconiti Lateralis Radix Praeparata, known for its warming yang properties and ability to transform dampness, is often prescribed for conditions related to cold and dampness 4. Additionally, Herba Patriniae is recognized for enhancing immune function and promoting the flow of qi and blood 5. In colorectal cancer, YYFZBJS has been shown to influence tumor progression by reducing Treg cell numbers in the intestinal and mesenteric lymph nodes of mice and by remodeling the intestinal microbiota 6. Another study demonstrated that YYFZBJS decreased the infiltration of M2 macrophages in the colon by reducing Bacteroides fragilis colonization, alleviating inflammation and improving colorectal tumor progression 7. Furthermore, YYFZBJS regulates the G2/M phase by inhibiting the CDK/PI3K/AKT signaling pathway in vivo and in vitro, inducing apoptosis and enhancing tumor progression 8. These studies underscore YYFZBJS's role in reshaping the intestinal microbiota, regulating immune responses, and promoting cell apoptosis. Given its substantial impact on various pathological processes related to tumor treatment, YYFZBJS holds promising clinical potential. However, its effects on NPC remain unexplored.

In this study, we employed network pharmacology analysis to identify the core compounds and targets of YYFZBJS in the treatment of NPC. The identified targets were validated using the GSE dataset, and the binding efficacy between core compounds and targets was assessed through molecular docking. Our integration of network pharmacology and bioinformatics analysis provided reliable data for investigating the effects of YYFZBJS on NPC, establishing a theoretical foundation for further experimental studies and clinical applications.

## Materials and methods

### Screening of active compounds and their corresponding targets of YYFZBJS

**Figure 1:**
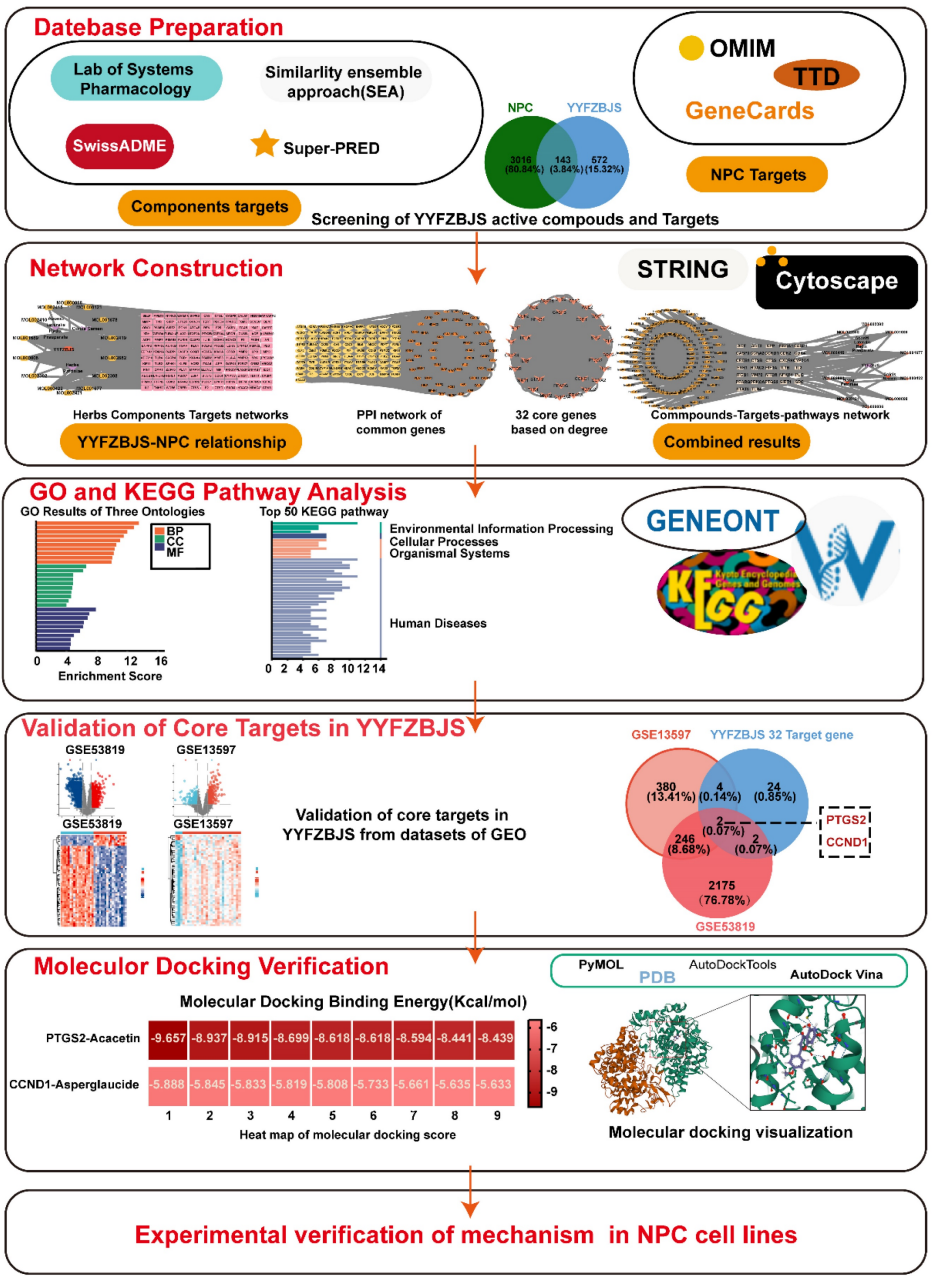
A detailed workflow of the network pharmacological investigation strategy for YYFZBJS in the treatment of NPC. Five parts include datebase preparation, GO and KEGG pathway analysis, network construction, validation of core targets, and molecular docking verification in YYFZBJS.

The TCM Systematic Pharmacology Database and Analysis Platform (TCMSP: http://tcmspw.com/tcmsp.php) was utilized to identify the active compounds and their interacting proteins for YYFZBJS. Two key pharmacokinetic parameters, oral bioavailability (OB) and drug-likeness (DL), were applied as screening criteria, with thresholds set at OB≥30% and DL≥0.18 9, 10. Additionally, The SMILES format of the active compounds was obtained from the PubChem database and subsequently imported into the SwissADME platform, where gastrointestinal absorption (GI) and drug-likeness were assessed. GI was assessed as high, and the drug-likeness criteria, which required at least two "yes" responses, identified the active ingredient of the herbal medicine. The SMILES format was then imported into the Similarity Ensemble Approach (SEA) database (https://sea.bkslab.org/) to identify the targets of the active ingredients. For the compound MOL002415-6-Demethyldesoline, which lacked data in the SEA database, potential targets were searched using the Super-PRED platform (https://prediction.charite.de). All identified targets were integrated, with duplicates removed, resulting in a comprehensive list of relevant targets. The study flowchart was presented in Fig. 1.

### Gathering of NPC-related targets

To identify potential targets for NPC, we searched across three online databases: OMIM (https://omim.org/), GeneCards: The Human Gene Database(https://www.genecards.org/), and the Therapeutic Target Database (TTD) (http://db.idrblab.net/ttd/). These databases provided comprehensive information on human genes, genetic diseases, and potential therapeutic targets. The identified targets were then integrated and duplicates removed to generate a complete list of relevant targets.

### The common targets overlapped between active compounds and NPC-related targets

The analysis identified shared targets between NPC-related targets and predicted YYFZBJS targets, which were visualized using a Venn diagram created with the tool. (http://www.bioinformatics.com.cn/static/others/jvenn/example.html).

### Constructing a network to visualize PPI

Genes identified as both drug and disease targets were imported into the STRING database (https://string-db.org/), a tool for exploring interactions among proteins and proteins. The database parameters were configured to focus on the 'human' species, with a protein-protein interaction (PPI) confidence score set at 0.4. Networks of potential key targets were generated using Cytoscape software (version 3.9.1). Additionally, a network of potential key targets was constructed using the CentiScape 2.2 tool, which automatically generated thresholds for three parameters: Degree Centrality (DC) ≥ 44.141843971631204, Closeness Centrality (CC) ≥ 0.0035515252329919953, and Betweenness Centrality (BC) ≥ 148.12765957446805, to identify core genes.

### GO and KEGG enrichment analyses

GO and KEGG analyses of the 32 core gene targets were performed using an online platform (http://www.bioinformatics.com.cn/), which integrated R packages, including cluster Profiler and pathview. The cutoff for GO analysis was set at a *P* value < 0.05, while the cutoff for KEGG analysis was established at an adj.*P* value < 0.05.

### Construction of topology network

To elucidate the connection between the active compounds of YYFZBJS and their corresponding "drug-disease" targets, we constructed a "drug-active compound-target" network using Cytoscape version 3.9.1 for topology analysis and visualization. Additionally, we used Cytoscape to build and analyze a "compound-target-pathway" network, which visually represented the complex interactions among compounds, targets, and pathways. This network provided deeper insights into the mechanisms of the compounds and facilitated the identification of potential drug targets and pathways.

### NPC datasets acquisition and target validation

Two datasets, GSE53819 and GSE13597, were retrieved from the Gene Expression Omnibus database (https://www.ncbi.nlm.nih.gov/geo/). The GSE53819 dataset included 18 primary tumors from NPC patients as the experimental group and 18 noncancerous nasopharyngeal tissues as the control group, all of which were diagnosed by pathology (**Supplementary Table 1**). In contrast, the GSE13597 dataset included nasopharyngeal tumors from 25 histologically confirmed undifferentiated NPC patients as the experimental group and tissues from 3 healthy volunteers as the control (**Supplementary Table 2**). We employed the 'limma' R software package for differential analysis, setting the threshold at |LogFC|≥ 1 and *P* < 0.05. Volcano plots and heat maps were generated using the 'ggplot2' and 'pheatmap' R packages, respectively. Venn diagrams were utilized to identify potential common targets among the 32 core genes of YYFZBJS with two NPC datasets, GSE53819 and GSE13597. Subsequently, the Cancer Genome Atlas (TCGA) database of head and neck tumors, comprising 44 adjacent cancer tissues and 504 head and neck tumor tissues, further confirmed that the intersecting genes identified in the aforementioned Venn diagram were present in HNSC containing NPC. Next, its relationship with 5-year survival rate and disease-free survival was analyzed.

### Molecular docking verification

Core ligand structure files (SDF) were retrieved from the PubChem database (https://pubchem.ncbi.nlm.nih.gov) and converted into PDB files using PyMOL version 2.5.5 (https://pymol.org/2/). The three-dimensional crystal structures of the targets were obtained from the RCSB Protein Data Bank (RCSB database: https://www.pdb.org/) and imported into PyMOL for ligand extraction. The ligand and receptor were then imported into AutoDockTools version 1.5.6 (https://ccsb.scripps.edu/mgltools/downloads/) for dehydration, hydrogenation, and charge calculation, after which they were saved in PDBQT format as a preparatory step for molecular docking. Subsequently, grid box for docking was constructed in AutoDockTools, encompassing the entire target protein, and the parameters were saved in text format. Finally, molecular docking analysis was conducted using AutoDock Vina version 1.1.2 (https://vina.scripps.edu), followed by thermal mapping using the 'ggplot2' and 'pheatmap' R packages to generate heat maps illustrating free binding energies. Molecular docking interactions were visualized using PyMOL.

### Molecular dynamics simulation

Molecular dynamics simulations were performed using Desmond/Maestro noncommercial version 2022.1 as the simulation software. TIP3P water molecules were added to the systems, which were then neutralized by 0.15 M NaCl solution. After system minimization and relaxation, a production simulation was conducted for 100 ns in an isothermal-isobaric ensemble at 300 K and 1 bar. Trajectory coordinates were recorded every 100ps. Molecular dynamics analysis was carried out using Simulation Interaction Diagram from Desmond.

### Cell culture

The 6-10B (C1651, WHELAB, Shanghai, China) cell lines were acquired from Shanghai Meiwan Biotechnology Co,while the C666(SNL-516, Wuhan, Sunncell, Wuhan, China) cell lines were sourced from Wuhan Sunncell Biotechnology Co. Both the 6-10B and C666 cells were cultured in RPMI-1640 (G4531, Servicebio, Wuhan, China) supplemented with fetal bovine serum (FBS) at a final concentration of 10%. The cells were maintained in an incubator under standard conditions. Cell passaging and seeding were performed when the cell coverage reached70%-85%. Subsequent cell experiments divided the cells into a control group and an experimental group treated with 50μM acacetin (HY-N0451, MedChemExpress, Shanghai, China).

### Cell counting kit-8

The acacetin-treated NPC cell lines and control cells were seeded into 96-well plates at 1000 cells per well. The medium was replaced with fresh medium containing 10% cell CCK-8 reagent (BS350A, Biosharp, Hefei, China) and the cells were incubated at 37°C for 1 h. Then, cell viability was assessed at 450 nm.

### Colony formation

The Acacetin-treated NPC cell lines and control cells were seeded into six-well plates at 500 cells per well. The cells were cultured according to the aforementioned treatment protocol for a duration of two weeks, or until colonies became visible to the naked eye. Subsequently, the cell colonies were fixed and stained with a 0.3% crystal violet ethanol solution for 15 minutes. Finally, the whole well was photographed.

### Transwell migration assay

The cell migration was evaluated using transwell inserts. The acacetin-treated NPC cell lines and control cells were seeded into the transwell inserts at a concentration of 5 × 10^^5^ cells per well and incubated overnight. Each assay was conducted in triplicate and subsequently stained with crystal violet.

### PCR assay

TRIzol reagent was used to extract total RNA, and cDNA synthesis was performed according to the protocol of the transcriptase kit (Vazyme Biotech, Nanjing, China). Quantitative reverse transcription polymerase chain reaction (qRT-PCR) was conducted on a CFX96 Connect instrument (Bio-Rad, Hercules, CA) and a SYBR Green PCR kit (Servicebio, Hubei, China). The mRNA expression levels were calculated by the 2^^-ΔΔCt^ method. The primer sequences are provided in the below: Forward primer: GTTCCACCCGCAGTACAGAA, Reverse primer: AGGGCTTCAGCATAAAGCGT.

### Western blot

The western blot assays were performed as previously described. Proteins were detected using the following antibodies: anti-PTGS2 antibodies (dilution, 1:1000; cat. A21131, Nature Biosciences, Hangzhou, Zhejiang), anti-ACTB (dilution 1:4000, 20536-1-AP, Proteintech, Wuhan, Hubei, China).

### Statistics

The data are expressed as mean ± standard deviation. Statistical comparisons were performed by Student's t test and analysis of variance with *P*<0.05 were considered significant.

## Results

### Candidate targets and their corresponding active compounds of YYFZBJS

**Figure 2:**
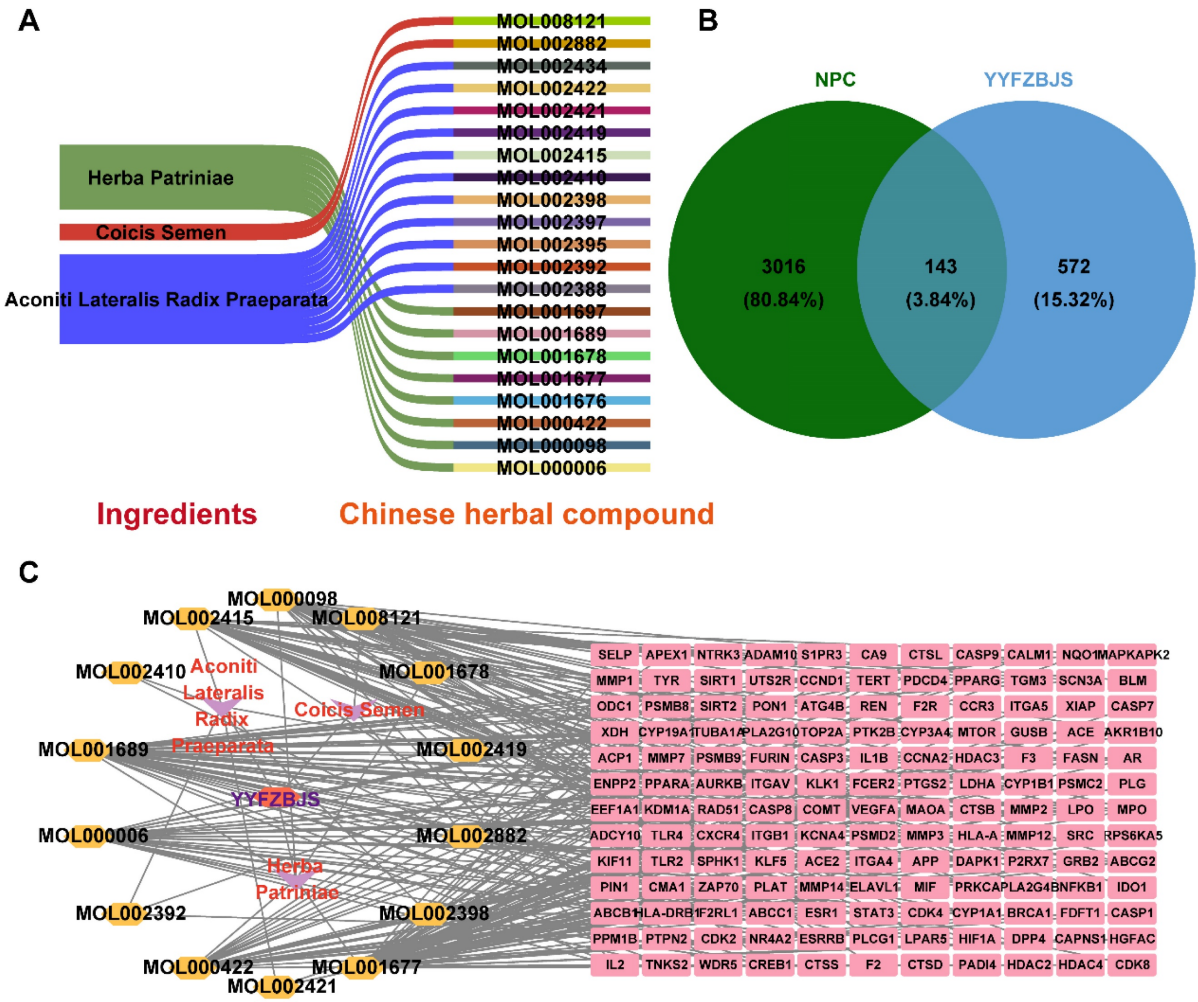
**Screening of YYFZBJS active compounds and targets in NPC. (A)** Sankey diagram of associations between Chinese herbal compounds and ingredients in YYFZBJS. **(B)** Venn diagram of 143 common targets between active compound of YYFZBJS and NPC disease Targets. **(C)** Herb-compound-target network.

Three herbal medicines derived from YYFZBJS were sourced from the TCMSP database. A total of 21 potent molecular compounds were identified based on their GI profiles and drug-likeness characteristics (**Supplementary Table 3**). To visualize the direct relationships between the herbal compounds and the ingredients in YYFZBJS, Sankey diagrams were generated using online sites (http://www.bioinformatics.com.cn/) (**Fig. 2A**).

A total of 715 potential targets associated with YYFZBJS, along with their corresponding gene symbols, were identified by importing the SMILES representations of 21 potent pharmaceutical active ingredients into the Swiss Target Prediction platform (http://swisstargetprediction.ch/), following the removal of duplicate entries (**Supplementary Table 4**).

NPC-related targets were collected were collected from GeneCards, OMIM, and TTD, and after duplicate entries were removed, a total of 3,159 targets associated with NPC disease were identified (**Supplementary Table 5**). A Venn diagram revealed 143 common targets shared between YYFZBJS active compounds and NPC, which may serve as potential therapeutic targets for YYFZBJS in treating NPC (**Fig. 2B, Table 1**). These common targets were promising candidates for YYFZBJS in the treatment of NPC.

The herb-compound-target network, which comprised three herbs, fourteen compounds, and 143 targets, was constructed using Cytoscape (**Fig. 2C**). The edges in the network illustrate the relationships among the herbs, compounds, and targets, indicating that the mechanism by which YYFZBJS treated NPC involved multiple compounds that target various biological pathways. Notably, the compounds 6-MOL002434-Carnosifloside I_qt, 7-MOL002388-Delphin_qt,9-MOL002395-Deoxyandrographolide, 11-MOL002422-Isotalatizidine, 12-MOL002397-Karakoline, 14-MOL001676-Vilmorrianine C, and 18-MOL001697-Sinoacutine exhibited no overlapping genes with the identified target genes, suggesting that these compounds may not be the primary active ingredients in NPC treatment.

### Analysis and construction of PPI network for common targets

**Figure 3:**
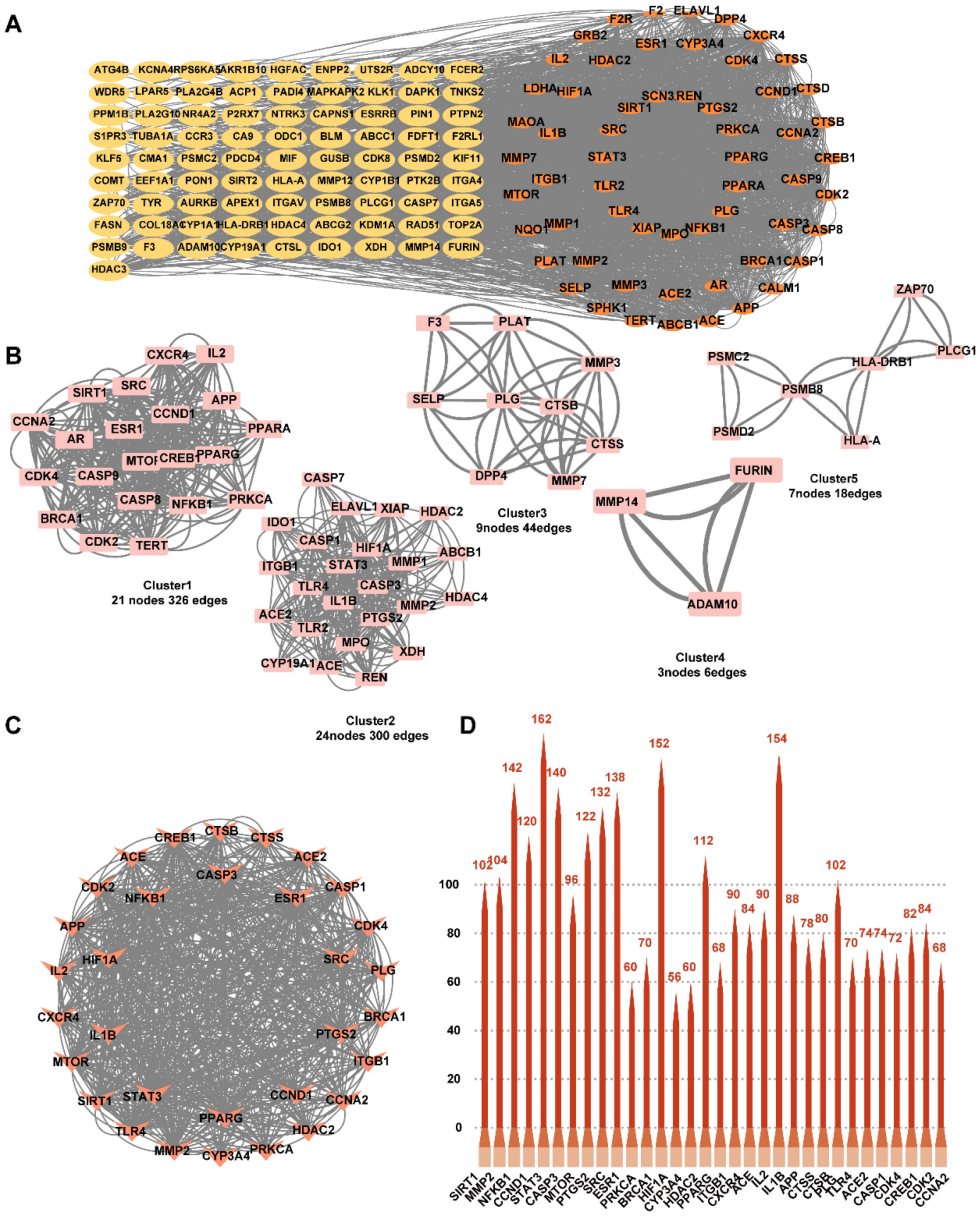
Identification of candidate targets through Protein-Protein Interaction (PPI) analysis. **(A)** PPI analysis. **(B)** PPI network based on cluster analysis using the MCODE plugin. **(C)** Identification of top 32 core targets based on DC≥22.070921985815602. **(D)** Bar chart of the top 32 core targets.

To investigate the mechanism of action of YYFZBJS on NPC, we constructed a PPI network based on 143 common targets utilizing STRING and Cytoscape (**Fig. 3A**). Three centrality metrics-DC, CC, and BC- were employed in the network analysis. Using these methods, we identified 32 core genes. Since DC serves as the most direct indicator of node centrality, it was prioritized for network mapping. Additionally, the MCODE plugin in Cytoscape was used for cluster analysis, resulting in the construction of five highly connected sub-networks. The target genes were classified into five clusters: Cluster 1 (21 nodes, 326 edges), Cluster 2 (24 nodes, 300 edges), Cluster 3 (9 nodes, 44 edges), Cluster 4 (3 nodes, 6 edges), and Cluster 5 (7 nodes, 18 edges), with score values of 16.30, 13.04, 5.50, 3.00, and 3.00, respectively. Higher score values indicated more significant sub-networks (**Fig. 3B**). Furthermore, based on the topology analysis screening criteria, the 32 core target genes with DC ≥ 22.070921985815602 were identified (**Fig. 3C, Table 2**). The top 32 core targets were represented as bar charts (**Fig. 3D**).

### GO and KEGG enrichment analysis

**Figure 4:**
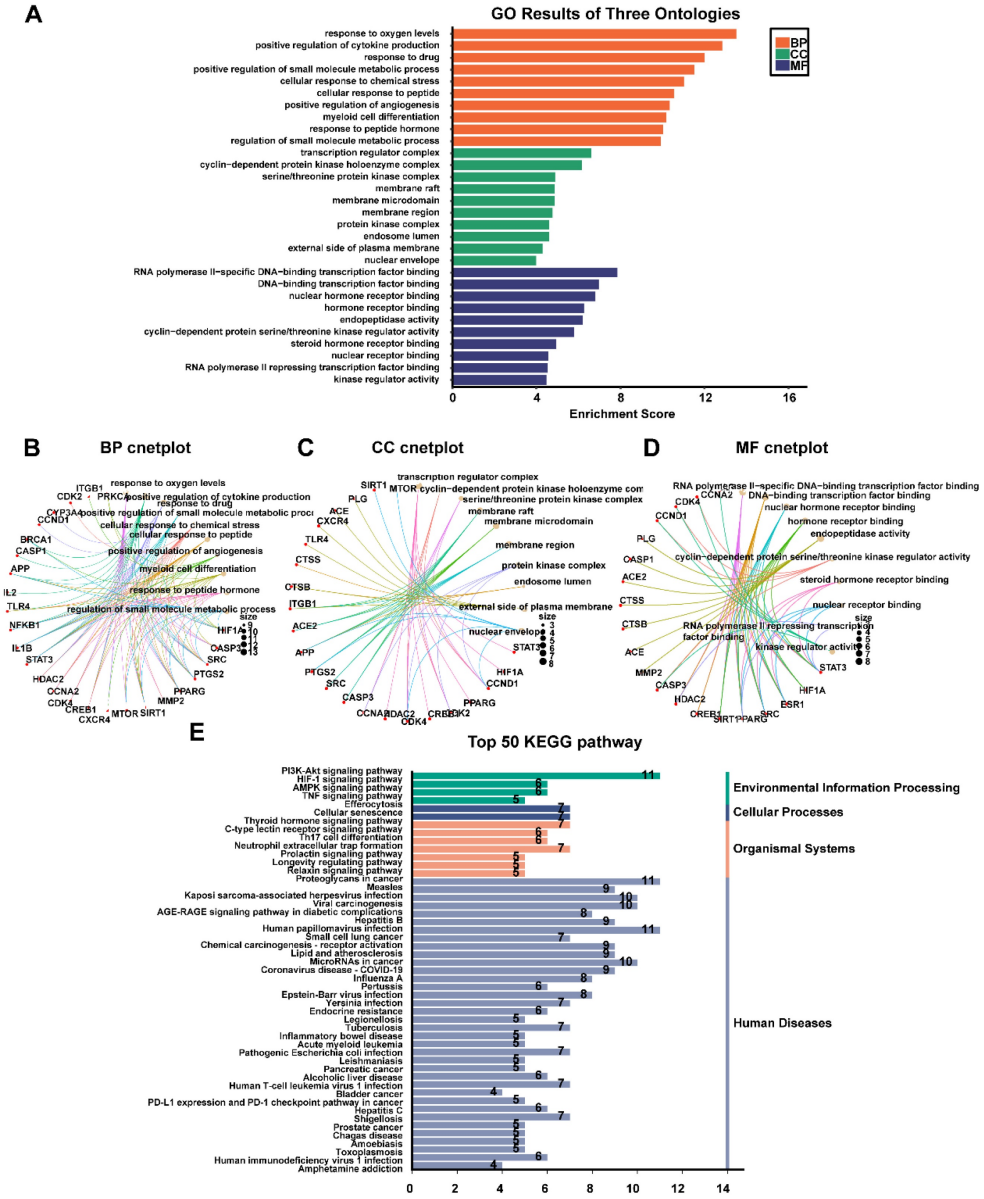
** Results of GO, and KEGG enrichment analysis. (A)** Barplot chart of GO functional enrichment analysis about BP, CC and MF. **(B)** BP cnetplot. **(C)** CC cnetplot. **(D)** MF cnetplot. **(E)** Barplot chart of the top 50 pathways based on KEGG enrichment analysis.

The top 32 "drug-disease" targets were analyzed for GO and KEGG enrichment using an online platform. The GO analysis yielded a total of 2052 terms, comprising 1827 biological processes (BPs), 85 cellular components (CCs), and 140 molecular functions (MFs) (**Fig. 4A, Supplementary Table 6**). The most significantly enriched BP terms were primarily associated with oxygen sensing, cytokine production, and drug response. The CC terms predominantly focused on membrane rafts, membrane microdomains, and the plasma membrane. Furthermore, the highly enriched MF terms included RNA polymerase II-specific DNA-binding transcription factor binding, general DNA-binding transcription factor binding, and nuclear hormone receptor binding. The 32 core genes involved in these pathways were illustrated in the circular maps (**Fig. 4B, 4C, 4D**). A total of 115 signaling pathways were identified in the top 50 KEGG-enriched analyses. Among the most enriched KEGG pathways, the top-ranked signaling pathways, including the PI3K-Akt signaling pathway, HIF-1 signaling pathway, AMPK signaling pathway, and TNF signaling pathway, were closely related to the development of NPC (**Fig. 4E, Supplementary Table 7**).

### Compound-target-pathway analysis

**Figure 5:**
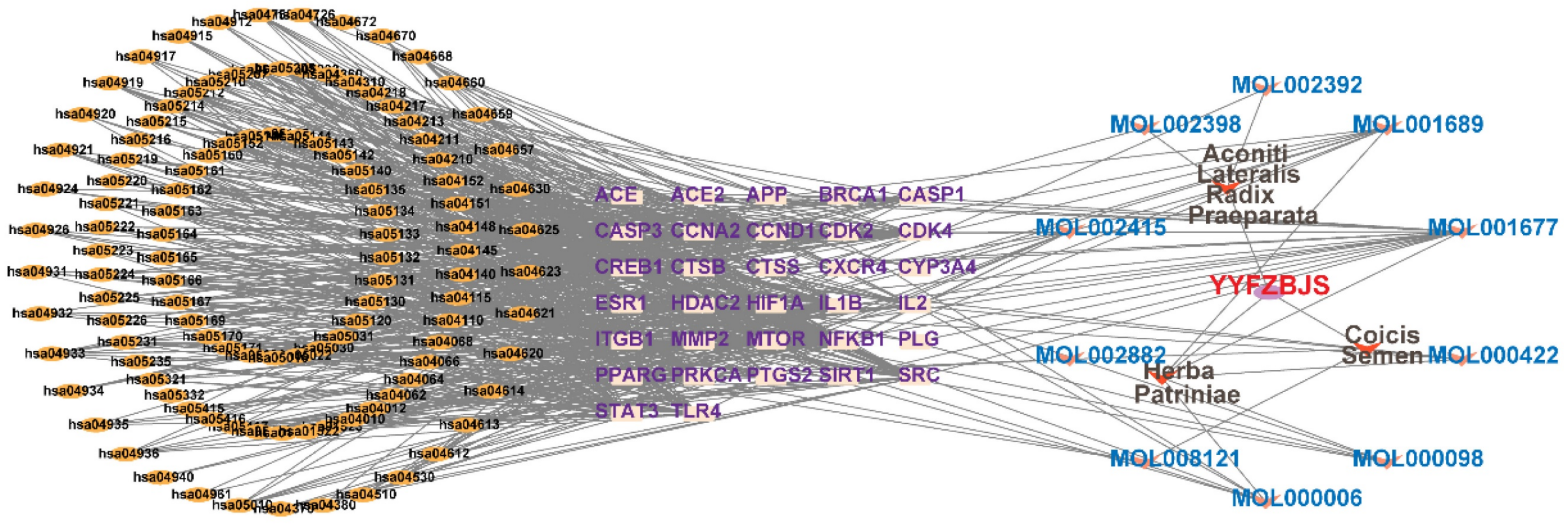
Construction of compound-target-pathway network.

The compounds MOL002419 (R)-Norcoclaurine, MOL002410-benzoylnapelline, MOL002421-Ignavine, and MOL001678-Bolusanthol B did not intersect with the 32 core genes. In contrast, the remaining 10 pharmacologically active ingredients were considered to have therapeutic potential for NPC. These compounds are MOL008121-2-Monoolein, MOL002882-[(2R)-2,3-dihydroxypropyl] (Z)-octadec-9-enoate, MOL002415-6-Demethyldesoline, MOL002392-Deltoin, MOL002398-Karanjin, MOL001677- Asperglaucide, MOL001689-Acacetin, MOL000422-Kaempferol, MOL000006-Luteolin, and MOL000098-Quercetin. Further details can be found in **Table 3**. These 10 compounds were used for subsequent network construction. Cytoscape was employed to construct a network of compound-core gene-pathway (**Fig. 5, Supplementary Table 8**).

### Validation of YYFZBJS related target genes using NPC data from the GEO database

**Figure 6:**
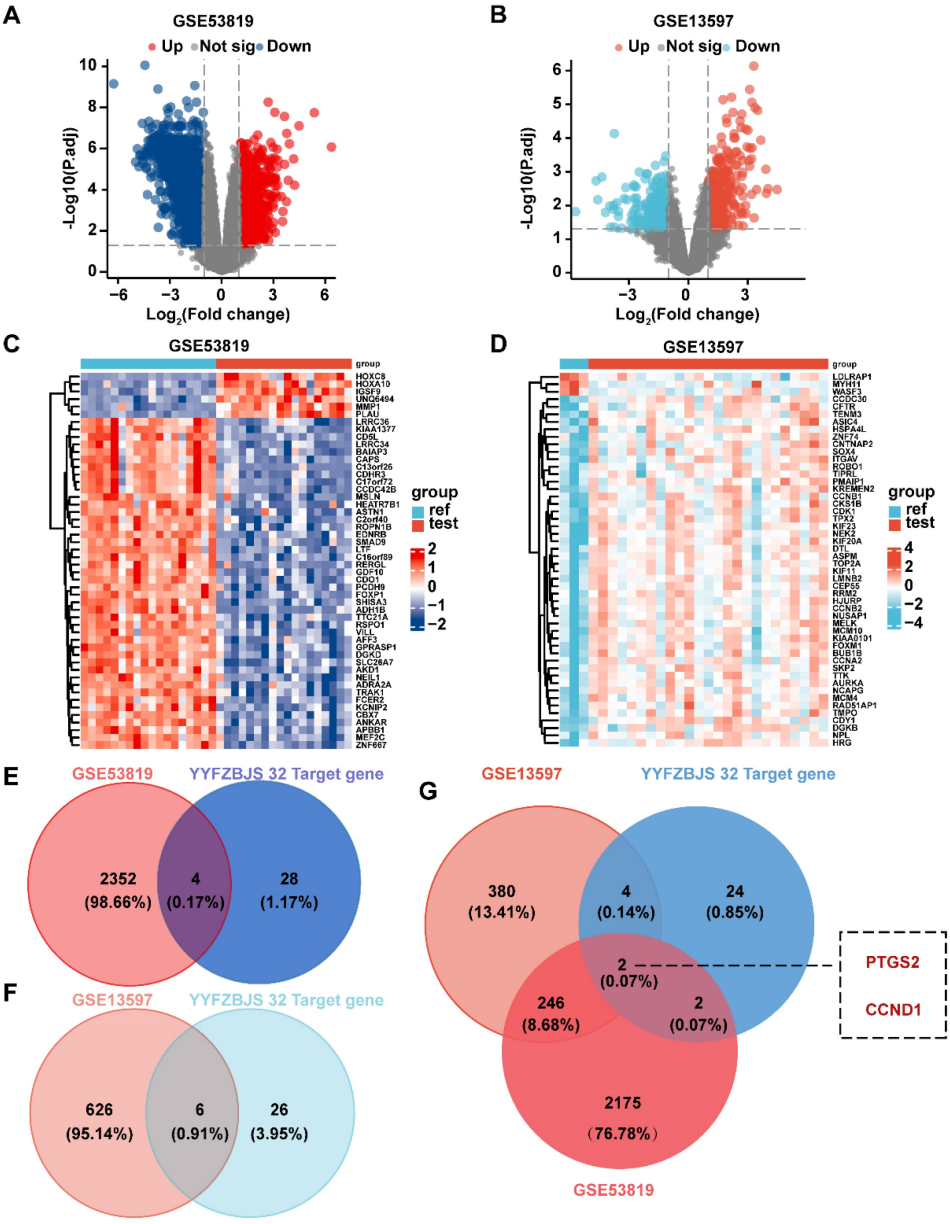
** Validation of 32 core targets in YYFZBJS from datasets of GEO (GSE53819 and GSE13597). (A)** Volcano map showing differential genes for GSE53819. **(B)** Volcano map showing differential genes for GSE13597. **(C)** The heat map of GSE53819. **(D)** The heat map of GSE13597. **(E)** Venn diagram showing intersecting targets of GSE53819 and 32 target genes of YYFZBJS.** (F)** Venn diagram showing intersecting targets of GSE13597 and 32 targets genes of YYFZBJS. **(G)** Venn analysis of core genes in the GSE53819, GSE13597 and 32 targets genes of YYFZBJS.

Two datasets from the GEO database, GSE53819 and GSE13597, were analyzed for differentially expressed genes using the 'limma' R package. The number of differentially expressed genes in GSE53819 was illustrated by a volcano plot (**Fig. 6A**), where red indicated the893 up-regulated genes and green signified the 1463 down-regulated genes. Similarly, the differential gene expression in GSE13597 was represented by a volcano plot (**Fig. 6B**), with red representing 393 up-regulated genes and blue representing 239 down-regulated genes. The distribution of differential genes in the 2 datasets was visualized by heatmaps (**Fig. 6C-D**).

To identify common core target genes between the drug targets and the GSE53819 dataset, an intersection analysis was conducted using a Venn diagram (**Fig. 6E**). This analysis revealed four common genes: PTGS2 (LogFC = 2.26, *P* < 0.05), CYP3A4 (LogFC = 1.84, *P* < 0.05), IL1B (LogFC = 1.63, *P* < 0.05), and CCND1 (LogFC = 1.35, *P* < 0.05). Additionally, six common target genes were identified between the drug targets and the GSE13597 dataset (**Fig. 6F**): PTGS2 (LogFC = 3.20, *P* < 0.05), CCNA2 (LogFC = 1.45, *P* < 0.05), PRKCA (LogFC = 3.20, *P* < 0.05), CCND1 (LogFC = 1.10, *P* < 0.05), CDK4 (LogFC = 1.07, *P* < 0.05), and HDAC2 (LogFC = 1.06,* P* < 0.05).Finally, the intersection between the drug targets and the two datasets was analyzed to identify the common core target, resulting in the identification of two central genes: PTGS2 and CCND1 (**Fig. 6G**). These genes were confirmed to be the most significant targets for the network pharmacological identification of YYFZBJS's action on NPC.

To further illustrate the significance of the two genes, we utilized TCGA database to investigate their individual roles. Our analysis revealed that PTGS2 was significantly elevated in head and neck tumors compared to normal tissues. This increased expression was notably associated with a decline in the patients' 5-year survival rates; however, no significant statistical difference was observed concerning disease-free survival. In contrast, the expression of CCND1 in head and neck tumors showed no significant variation, but its elevated expression correlated with the 5-year survival rate, while it did not influence disease-free survival (**Fig. S1**). This analysis highlighted that PTGS2 may play a more important role than CCND1 in tumors.

### Validation and visualization of molecular docking results

**Figure 7:**
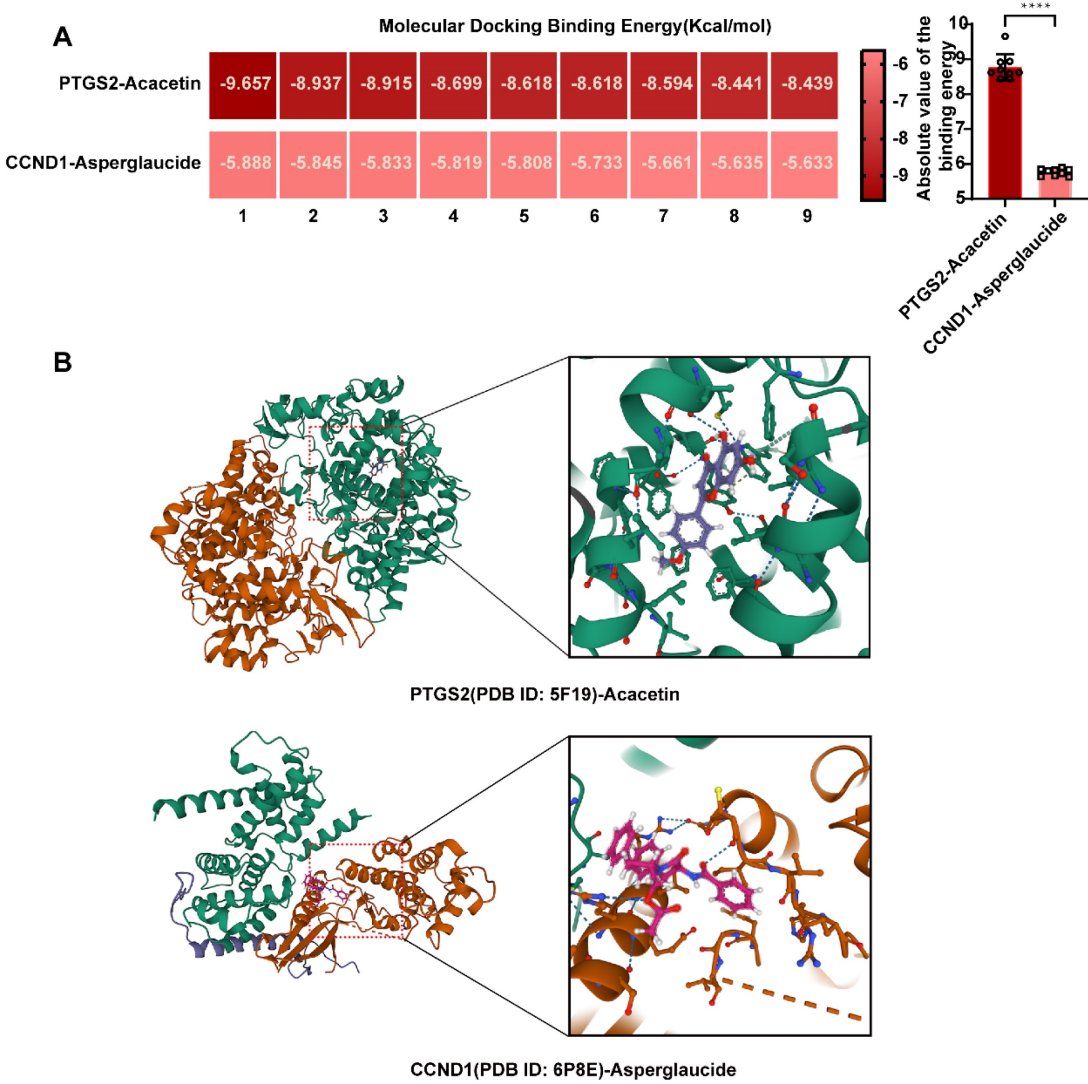
** Validation and screening of molecular docking. (A)** Heatmap of PTGS2-Acacetin and CCND1-Asperglaucide of NPC molecular docking scores. **(B)** Docking patterns of core targets and active compounds of NPC.

Through network pharmacology combined with microarray sequencing results from clinical samples, we identified PTGS2 and CCND1 as the relevant targets of YYFZBJS for the treatment of NPC. Subsequently, molecular docking was then performed between the active ingredients of YYFZBJS and the target proteins PTGS2 (PDB ID: 5F19) and CCND1 (PDB ID: 6P8E) to evaluate ligand-receptor interactions. The receptor proteins for molecular docking were obtained from the RCSB database. The binding energies, displayed in the heatmap, indicated that a binding energy significantly lower than -7Kcal/mol suggested strong binding between the target proteins and the compounds. Our molecular docking results revealed two ligand-receptor pairs, with notably strong binding activity between PTGS2 and Acacetin, while the interaction between CCND1 and Asperglaucide exhibited good binding activity (**Fig. 7A**). These findings implied that PTGS2 may serve as a more effective binding target for YYFZBJS in the treatment of NPC compared to CCND1. We selected the conformational docking positions with the lowest ligand-receptor binding energy for visualization using PyMOL, which allowed us to elucidate the interaction and binding patterns of the compounds and targets that displayed high free binding energy scores (**Fig. 7B**). The interactions between both target proteins and the compounds maintained stable ligand-receptor conformations through hydrogen bonding.

### Molecular dynamic

**Figure 8:**
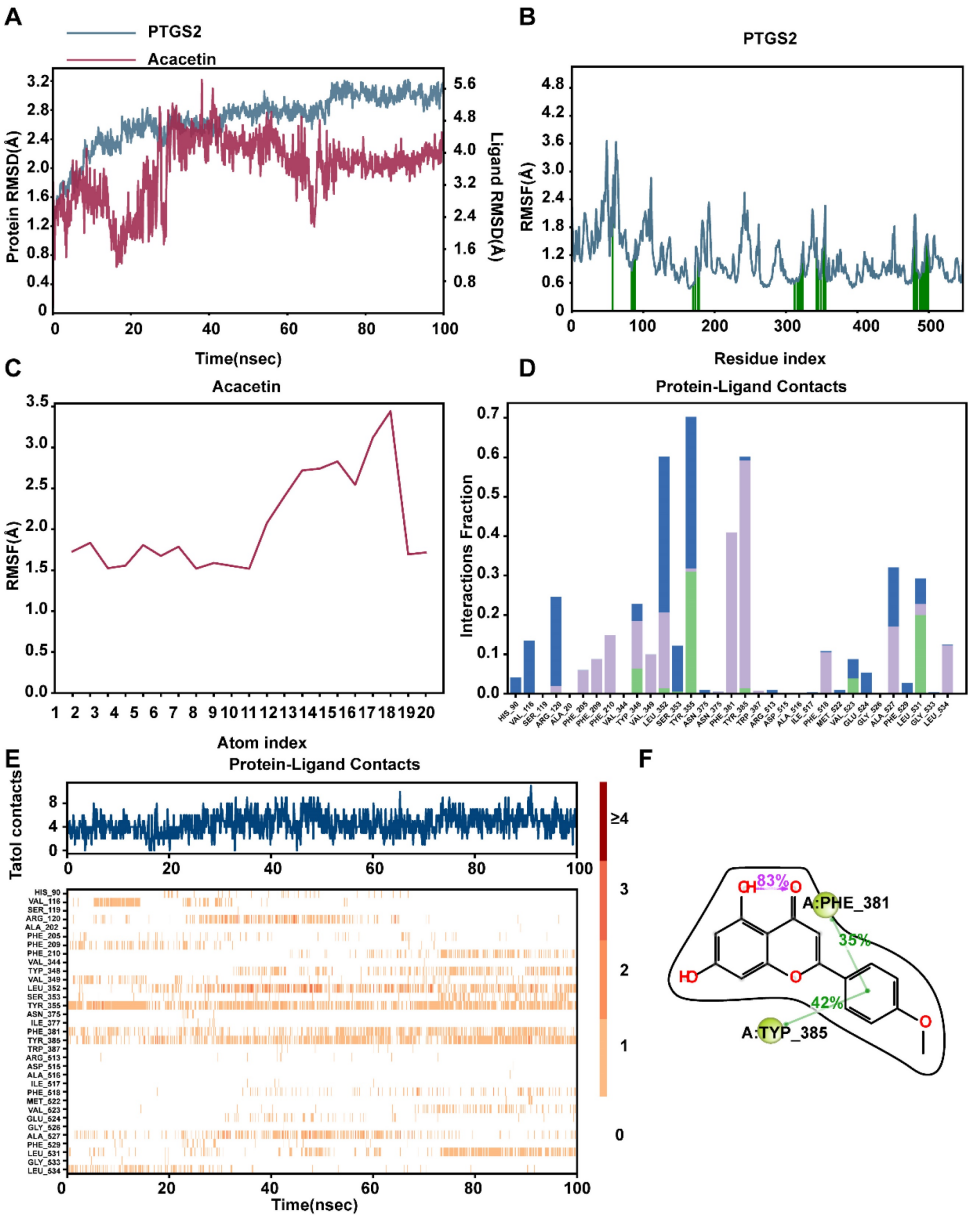
** Molecular dynamics simulation to predict ligand-protein for PTGS2 with acacetin (Simulation time 100 ns). (A)** RMSD analysis of PTGS2 and acacetin molecular dynamics simulation. **(B)** RMSF of PTGS2. **(C)** RMSF of Acacetin. **(D)** The bar graphs of PTGS2-acacetin contacts. **(E)** The timeline of PTGS2-acacetin contacts.

To evaluate the stability of the PTGS2-acacetin ligand complex and the structural flexibility of the protein, we utilized the Desmond/Maestro molecular dynamics software suite. Following the generation of Molecular Dynamics Simulation (MD) trajectories, we assessed the stability of the simulated PTGS2 and acacetin by calculating the root mean square deviation (RMSD) and the root mean square fluctuation (RMSF) of Cα atoms (**Fig. 8A-C**). The RMSD of all MD trajectories reached equilibrium around 100 ns, indicating comparable stability across all systems. The protein-ligand contacts observed during the MD simulations reveal that the PTGS2 protein and the acacetin small molecule formed multiple interactions (**Fig. 8D**). For instance, the frequency of hydrophobic interactions involving TYR385 was found to be 42%, suggesting that the TYR385 amino acid played a critical role in the binding process. Additionally, other hydrogen bonds and water bridges were formed, facilitating the interaction between the two (**Fig. 8E**). Furthermore, the ligand also established an intramolecular hydrogen bond, which stabilized the binding conformation of the small molecule (**Fig. 8F**). Preliminary results indicated that acacetin held significant potential as a mimetic compound for PTGS treatment.

### Acacetin inhibited NPC cell lines by downregulating the expression of PTGS2 *in vitro*

**Figure 9:**
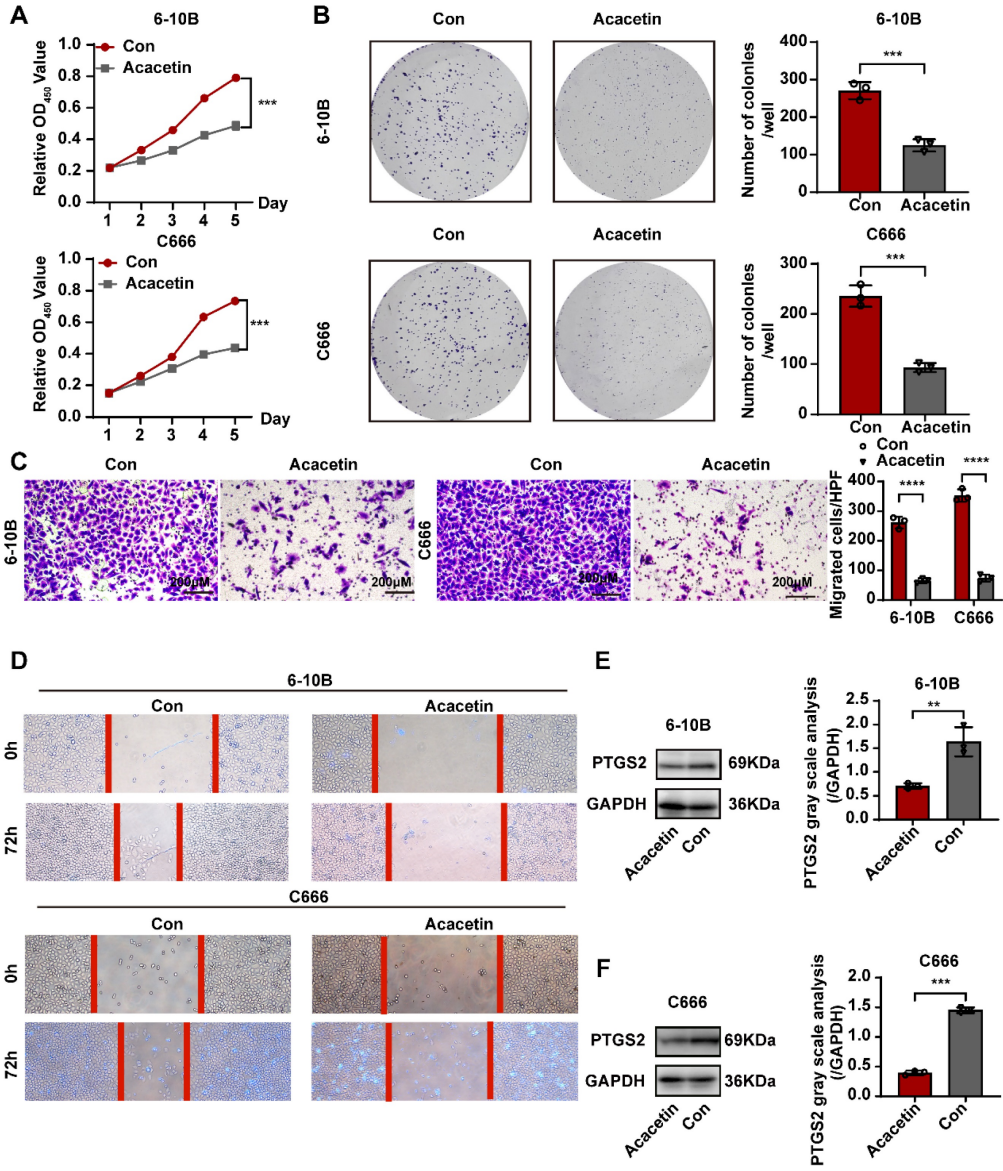
** Acacetin inhibited proliferation and migration of NPC cell lines by downregulating PTGS2 protein expression. (A)** CCK8 assay showed the relative proliferation ability of NPC cell lines(n=3). **(B)** Representative images of the wells of plates in the colony formation assay. The statistical analysis was displayed on the side(n=3). **(C)** Transwell migrated assay in NPC cell lines(n=3). The statistical analysis was displayed on the side.** (D)** Scratching wound-healing assay was performed to detect cell migration at 0h and 48h on control group and acacetin treated group. **(E, F)** The western blot confirmed PTGS2 protein expression was down-regulated after acacetin treatment. The statistical analysis was displayed on the side(n=3). **P < 0.01; ***P < 0.001; ****P <0.0001.

**Figure 10:**
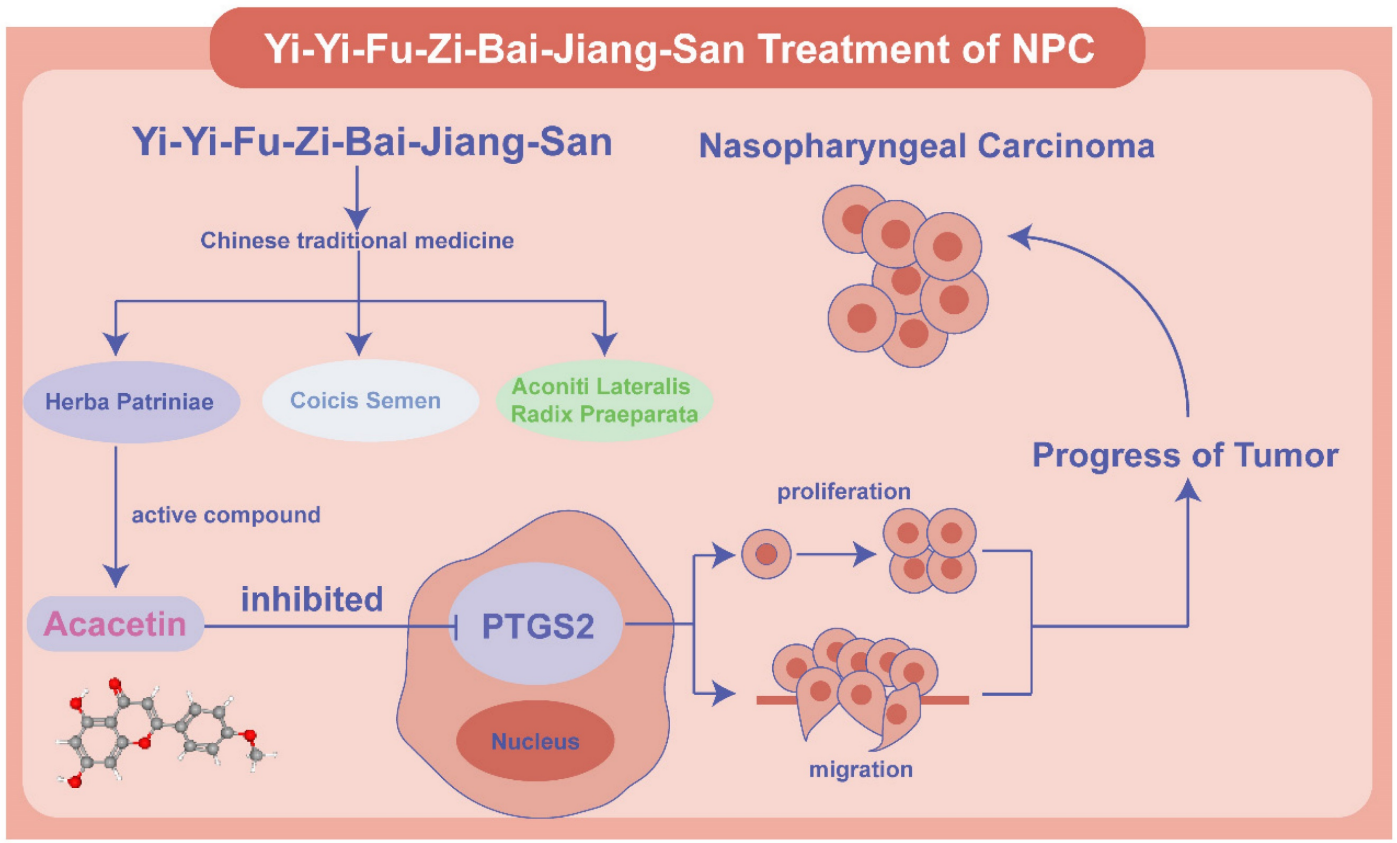
** Schematic diagram of the mechanism of Acacetin regulating PTGS2 in NPC.** Acacetin of the active ingredient in YYFZBJS inhibited the proliferation and migration of NPC cells by down-regulating PTGS2 expression.

Based on previous research, we determined that acacetin's ability to suppress the malignant phenotypic behavior of NPC cells was associated with the expression of PTGS2. To verify this hypothesis, we performed a series of cellular experiments for validation. The CCK8 assay and plate colony formation assay demonstrated that treatment with 50μM acacetin significantly reduced the proliferation of NPC cell lines compared to the control group **(Fig. 9A-B)**. Additionally, migration assays and scratch wound healing experiments indicated that the migratory capacity of acacetin-treated NPC cell lines was markedly diminished **(Fig. 9C-D)**. Furthermore, the western blot analysis revealed that acacetin significantly downregulated the expression levels of PTGS2 protein in NPC cell lines **(Fig. 9E-F)**. These results suggested that acacetin can effectively inhibit the proliferation and migration of NPC cell lines by downregulating PTGS2 protein, with the detailed mechanism pathway was shown in **Fig 10**.

## Discussion

The integration of TCM with radiotherapy has gained increasing attention in cancer treatment, particularly for NPC and other tumors 11. Combining of Chinese herbal preparations with radiotherapy has shown potential synergistic effects in clinical practice, improving therapeutic outcomes, reducing side effects, enhancing immune function, and promoting better overall quality of life. Studies indicate that specific active ingredients in TCM enhance the effects of radiotherapy through antioxidative mechanisms 12, promotion of apoptosis 13, and inhibition of cell proliferation 14. Additionally, TCM components can bolster patients' immune functions 15 and counteract radiotherapy-induced immunosuppression 16. These compounds also alleviate gastrointestinal reactions 17 and improve blood indices 18, mitigating other radiotherapy-related side effects. Current therapeutic options for NPC include small-molecule inhibitors, natural compounds, and TCM 19-21. YYFZBJS, a classic herbal formula, is recognized for its anti-tumor properties. In a population-controlled study of 2,469 newly diagnosed NPC patients and 2,559 control, compounds such as Coicis Semen were inversely associated with the development of NPC (OR = 0.63, *P* < 0.00) 22. We investigated the mechanism of YYFZBJS in NPC tumors using network pharmacology and bioinformatics analyses, offering new insights into the TCM concept of 'tonifying and eliminating diarrhea' in NPC.

In our study, 21 active compounds of YYFZBJS were screened, identifying 715 corresponding targets from the TCMSP database. We obtained 3,159 NPC-related targets from the GeneCards, OMIM, and TTD databases. The identification of 143 potential YYFZBJS-NPC targets enabled the construction and visualization of a PPI network, revealing 32 core targets based on DC. Subsequent GO and KEGG enrichment analyses identified the major biological processes and signaling pathways involved. Our KEGG pathway enrichment analysis indicated significant activation of the PI3K-AKT, HIF-1A, AMPK, and TNF signaling pathways. Activation of the PI3K-AKT pathway not only lowers NPC's susceptibility to radiotherapy but also promotes epithelial-mesenchymal transition, contributing to NPC metastasis 23. Therefore, PI3K-AKT signaling is crucial in both radiotherapy resistance and metastasis. Additionally, HIF-1A activation supports NPC adaptation to hypoxic environments, promoting malignant proliferation. Studies have shown that the LMP1 protein of Epstein-Barr virus upregulates HIF-1A expression, further enhancing NPC proliferation 24. Furthermore, targeting the AMPK-ULK1 signaling pathway induces autophagy, inhibiting tumor growth in NPC cells 25. High levels of TNF-α correlate with poor prognosis in NPC, with elevated serum TNF-α levels associated with significantly reduced 5-year survival rates, suggesting its potential as a prognostic biomarker 26. Our findings suggest that the active components of YYFZBJS may exert therapeutic effects on NPC by modulating these pathways.

We validated our findings by integrating data from the GSE database of NPC clinical samples, identifying two target-small molecule interactions-PTGS2-Acacetin and CCND1-Asperglaucide—as potentially the most effective therapeutic targets for NPC. Coicis Semen and Aconiti Lateralis Radix Praeparata, key herbal constituents of YYFZBJS, have drawn significant attention in oncology. For instance, Coicis Semen enhances the antitumor efficacy of PD-1 inhibitors in the A549 human non-small cell lung cancer cell line by inhibiting the PI3K-AKT-mTOR pathway 27.

Studies suggest that coixol, a compound in Coicis Semen, mediates its antitumor effects 28. Coicis Semen also inhibit lung carcinogenesis in mice by disrupting the Wnt/β-catenin signaling pathway 29. Moreover, the active compounds in Aconiti Lateralis Radix Praeparata exhibit anticancer properties, potentially through NF-κB, EMT, HIF-1, p38 MAPK, and PI3K/AKT/mTOR 4. While these findings suggest tumor-suppressive effects of both herbs, our bioinformatics analysis, incorporating network pharmacology and clinical NPC data from the GSE database, found no overlapping genes between the active ingredient targets and differentially expressed genes in NPC samples. This indicates that Coicis Semen and Aconiti Lateralis Radix Praeparata may not be central to NPC treatment based on current NPC-related network pharmacology analysis, highlighting complexity of drug targeting in various tumor-associated diseases.

Herba Patriniae, the third herbal component of YYFZBJS, has demonstrated anti-tumor effects in colorectal and bladder cancers through the P53^30^, TGF-β-Smad2/3-E-cadherin 5, and MAPK signaling pathways 31, supporting its role as a tumor suppressor. Our network pharmacology analysis, combined with clinical bioinformatics, revealed that Acacetin and Asperglaucide—the pharmacologically active components—interact with PTGS2 and CCND1 in clinical NPC tissue samples.

PTGS2 enhances tumor cell proliferation 32, promotes angiogenesis 33, regulates immune surveillance 34, and induces a pro-inflammatory tumor microenvironment 35. Elevated PTGS2 expression is an independent prognostic indicator for NPC, and inhibiting PTGS2 expression effectively suppresses NPC progression 36-38. Based on these findings, we hypothesize that Acacetin modulates NPC progression by influencing PTGS2 expression and its associated TNF signaling pathway, IL-17 signaling pathway, and NF-κB signaling, as suggested by network pharmacology analyses. Molecular docking and dynamics simulations confirmed the interaction between Acacetin and PTGS2, providing a solid foundation for further experimental validation.

CCND1, a member of the cell cycle regulatory proteins family, is primarily involved in the regulating of the G0/G1 phase. Abnormal CCND1 expression is observed in various cancers and correlates with poor prognosis in tumors, such as lymphoma 39 and melanoma 40. A similar pattern is observed in NPC, where CCND1 inhibition enhances the sensitivity to cisplatin 41, 42, indicating that targeting CCND1 could serve as a potential therapeutic strategy for this malignancy. Our findings suggest that Asperglaucide may inhibit tumor development by interacting with CCND1 to regulate key signaling pathways, including the AGE-RAGE signaling pathway, the PI3K-Akt signaling pathway, and the AMPK signaling pathway. Our molecular docking results showed that the Acacetin-PTGS2 (binding energy < -7 kcal/mol) has a stronger interaction than Asperglaucide-CCND1(binding energy > -7 kcal/mol), suggesting that Acacetin may exhibit stronger biological activity and targeting effects. Based on these results, Acacetin-PTGS2 emerges as a potential therapeutic strategy for NPC. Our previous studies have shown that YYFZBJS inhibits NPC tumor growth. However, the underlying mechanisms remain unclear due to the complexity of TCM components. Our analysis suggests that Acacetin-PTGS2 represents the most promising therapeutic component of YYFZBJS. Our results indicate that Acacetin significantly inhibits NPC cell proliferation and migration, reducing the malignant behavior of the tumor. Moreover, Acacetin effectively reduces PTGS2 expression in NPC cell lines, supporting the hypothesis that Acacetin in YYFZBJS suppresses NPC malignancy by inhibiting PTGS2 expression.

## Conclusion

Our study is the first to employ bioinformatics methods, including network pharmacology and molecular docking, to conduct a comprehensive investigation into the pharmacology and molecular mechanisms of YYFZBJS in the treatment of NPC. The results derived from bioinformatics and computational analyses were validated using two clinical GSE datasets, thereby enhancing the credibility of our findings. Ultimately, we identified Acacetin and Asperglaucide as the most effective pharmacologically active ingredients in YYFZBJS for the treatment of NPC, suggesting that they may exert anti-tumor effects through the modulation of PTGS2 and CCND1 expression. Moreover, we verify the effectiveness of Acacetin-PTGS2 as a potential approach to treat NPC in vitro. In summary, this study underscores the multi-component and pathway characteristics of YYFZBJS and elucidates its mechanism of action. These findings are anticipated to provide a solid theoretical foundation for the application of YYFZBJS in NPC and for the development of future pharmaceuticals.

## Supplementary Material

Supplementary figure.

Supplementary tables.

## Figures and Tables

**Table 1:** The 143 common targets between active compound of YYFZBJS and NPC disease.

NO.	Target	NO.	Target	NO.	Target	NO.	Target	NO.	Target	NO.	Target	NO.	Target
1	ABCB1	40	CYP1A1	79	LDHA	95	NR4A2	109	PPARG	123	SCN3A	141	XDH
2	ABCC1	41	CYP1B1	80	LPAR5	96	NTRK3	110	PPM1B	124	SELP	142	XIAP
3	ABCG2	42	CYP3A4	81	LPO	97	ODC1	111	PRKCA	125	SIRT1	143	ZAP70
4	ACE	43	DAPK1	82	MAOA	98	P2RX7	112	PSMB8	126	SIRT2		
5	ACE2	44	DPP4	83	MAPKAPK2	99	PADI4	113	PSMB9	127	SPHK1		
6	ACP1	45	EEF1A1	84	MIF	100	PDCD4	114	PSMC2	128	SRC		
7	ADAM10	46	ELAVL1	85	MMP1	101	PIN1	115	PSMD2	129	STAT3		
8	ADCY10	47	ENPP2	86	MMP12	102	PLA2G10	116	PTGS2	130	TERT		
9	AKR1B10	48	ESR1	87	MMP14	103	PLA2G4B	117	PTK2B	131	TGM3		
10	APEX1	49	ESRRB	88	MMP2	104	PLAT	118	PTPN2	132	TLR2		
11	APP	50	F2	89	MMP3	105	PLCG1	119	RAD51	133	TLR4		
12	AR	51	F2R	90	MMP7	106	PLG	120	REN	134	TNKS2		
13	ATG4B	52	F2RL1	91	MPO	107	PON1	121	RPS6KA5	135	TOP2A		
14	AURKB	53	F3	92	MTOR	108	PPARA	122	S1PR3	136	TUBA1A		
15	BLM	54	FASN	93	NFKB1	109	PPARG	123	SCN3A	137	TYR		
16	BRCA1	55	FCER2	94	NQO1	110	PPM1B	124	SELP	138	UTS2R		
17	CA9	56	FDFT1	95	NR4A2	111	PRKCA	125	SIRT1	139	VEGFA		
18	CALM1	57	FURIN	96	NTRK3	112	PSMB8	126	SIRT2	140	WDR5		
19	CAPNS1	58	GRB2	97	ODC1	113	PSMB9	127	SPHK1	141	XDH		
20	CASP1	59	GUSB	98	P2RX7	114	PSMC2	128	SRC	142	XIAP		
21	CASP3	60	HDAC2	99	PADI4	115	PSMD2	129	STAT3	143	ZAP70		
22	CASP7	61	HDAC3	100	PDCD4	116	PTGS2	130	TERT	123	SCN3A		
23	CASP8	62	HDAC4	101	PIN1	117	PTK2B	131	TGM3	124	SELP		
24	CASP9	63	HGFAC	79	LDHA	118	PTPN2	132	TLR2	125	SIRT1		
25	CCNA2	64	HIF1A	80	LPAR5	119	RAD51	133	TLR4	126	SIRT2		
26	CCND1	65	HLA-A	81	LPO	95	NR4A2	109	PPARG	127	SPHK1		
27	CCR3	66	HLA-DRB1	82	MAOA	96	NTRK3	110	PPM1B	128	SRC		
28	CDK2	67	IDO1	83	MAPKAPK2	97	ODC1	111	PRKCA	129	STAT3		
29	CDK4	68	IL1B	84	MIF	98	P2RX7	112	PSMB8	130	TERT		
30	CDK8	69	IL2	85	MMP1	99	PADI4	113	PSMB9	131	TGM3		
31	CMA1	70	ITGA4	86	MMP12	100	PDCD4	114	PSMC2	132	TLR2		
32	COMT	71	ITGA5	87	MMP14	101	PIN1	115	PSMD2	133	TLR4		
33	CREB1	72	ITGAV	88	MMP2	102	PLA2G10	116	PTGS2	134	TNKS2		
34	CTSB	73	ITGB1	89	MMP3	103	PLA2G4B	117	PTK2B	135	TOP2A		
35	CTSD	74	KCNA4	90	MMP7	104	PLAT	118	PTPN2	136	TUBA1A		
36	CTSL	75	KDM1A	91	MPO	105	PLCG1	119	RAD51	137	TYR		
37	CTSS	76	KIF11	92	MTOR	106	PLG	120	REN	138	UTS2R		
38	CXCR4	77	KLF5	93	NFKB1	107	PON1	121	RPS6KA5	139	VEGFA		
39	CYP19A1	78	KLK1	94	NQO1	108	PPARA	122	S1PR3	140	WDR5		

**Table 2:** 32 targets, ranked by degree in the compound-target-pathway network of NPC.

Gene name	Degree	Gene name	Degree
STAT3	162	APP	88
IL1B	154	ACE	84
HIF1A	152	CDK2	84
NFKB1	142	CREB1	82
CASP3	140	CTSB	80
ESR1	138	CTSS	78
SRC	132	ACE2	74
PTGS2	122	CASP1	74
CCND1	120	CDK4	72
PPARG	112	BRCA1	70
MMP2	104	PLG	70
SIRT1	102	ITGB1	68
TLR4	102	CCNA2	68
MTOR	96	PRKCA	60
CXCR4	90	HDAC2	60
IL2	90	CYP3A4	56

**Table 3:** Details of the 10 compounds in the compound-target-pathway network of NPC

Rank	Component ID	Component name	Structure	CAS	OB	DL
1	MOL008121	2-Monoolein	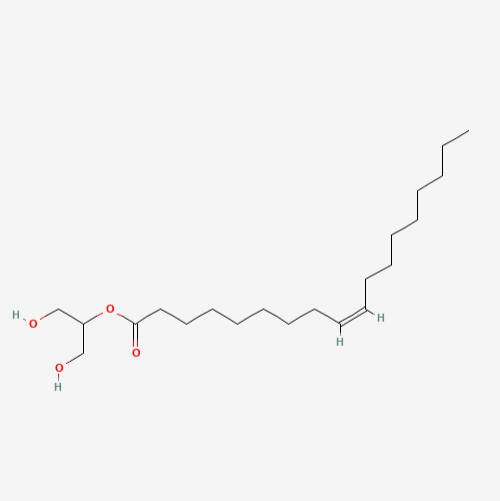	3443-84-3	34.23	0.29
2	MOL002882	[(2R)-2,3-dihydroxypropyl] (Z)-octadec-9-enoate	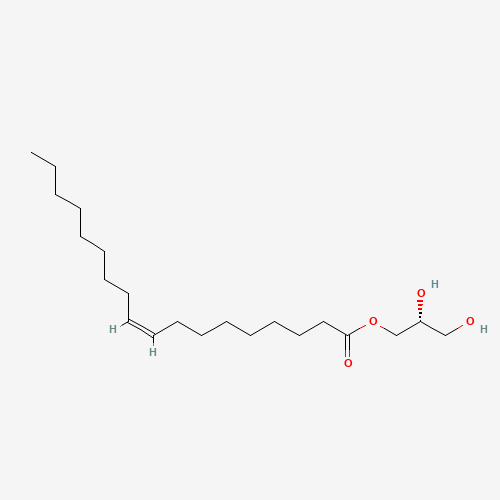	111-03-5	34.13	0.3
3	MOL002415	6-Demethyldesoline	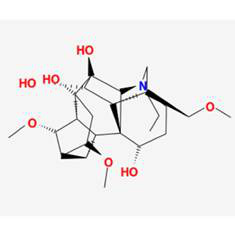	NA	51.87	0.66
4	MOL002392	Deltoin	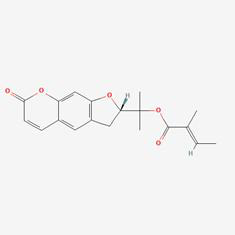	19662-71-6	46.69	0.37
5	MOL002398	Karanjin	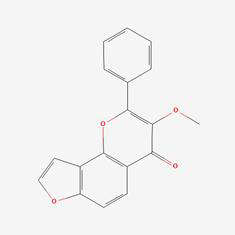	521-88-0	69.56	0.34
6	MOL001677	Asperglaucide	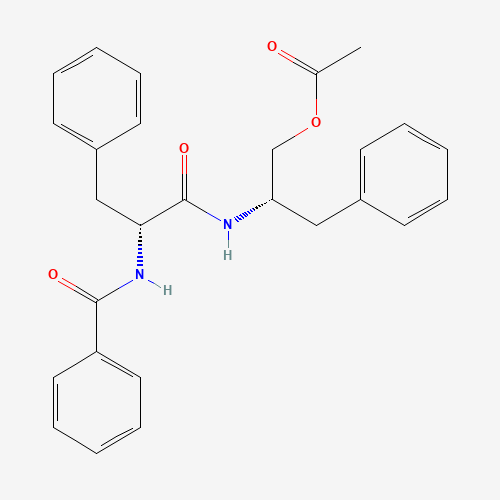	56121-42-7	58.02	0.52
7	MOL001689	Acacetin	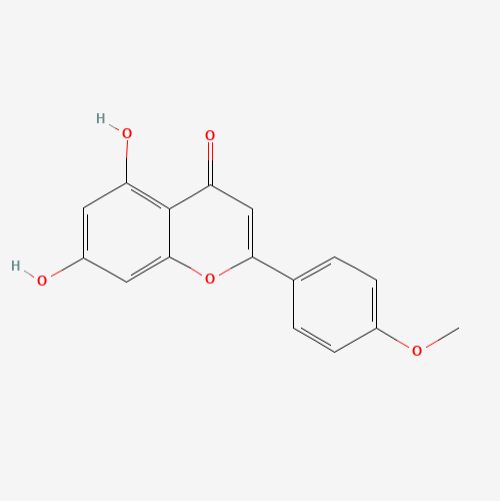	480-44-4	34.97	0.24
8	MOL000422	Kaempferol	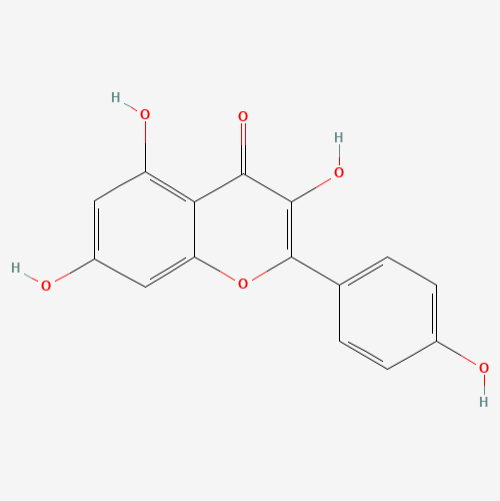	520-18-3	41.88	0.24
9	MOL000006	Luteolin	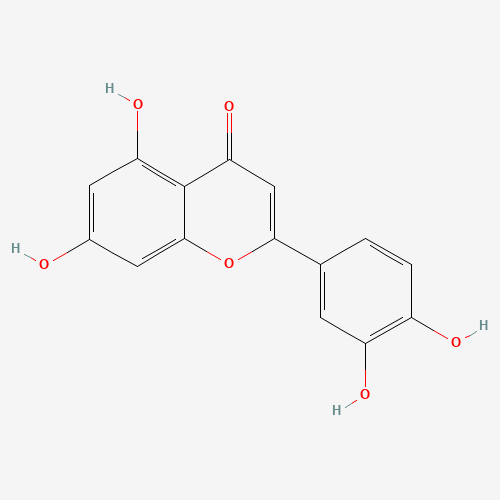	491-70-3	36.16	0.25
10	MOL000098	Quercetin	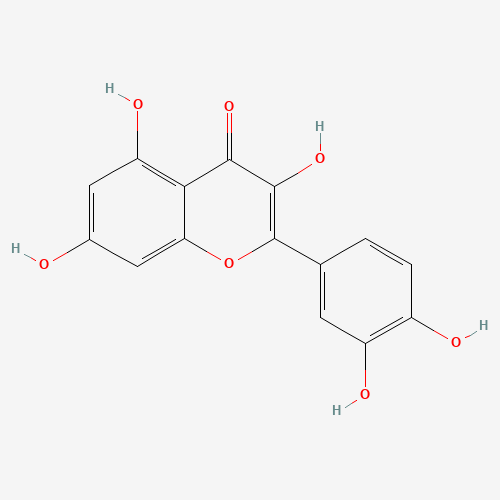	73123-10-1	46.43	0.28
