# Supplementary material for: Network Pharmacology and Experimental Validation-based Investigation of the Underlying Mechanism of Yi-Yi-Fu-Zi-Bai-Jiang-San of Nasopharyngeal Carcinoma
Source: J Cancer. 2025 Mar 29;16(7):2212–32. doi: 10.7150/jca.109758 (PMC12036090; doi:10.7150/jca.109758)
Supplement: Supplementary file 1 — Supplementary figure. [file jcav16p2212s1.pdf]

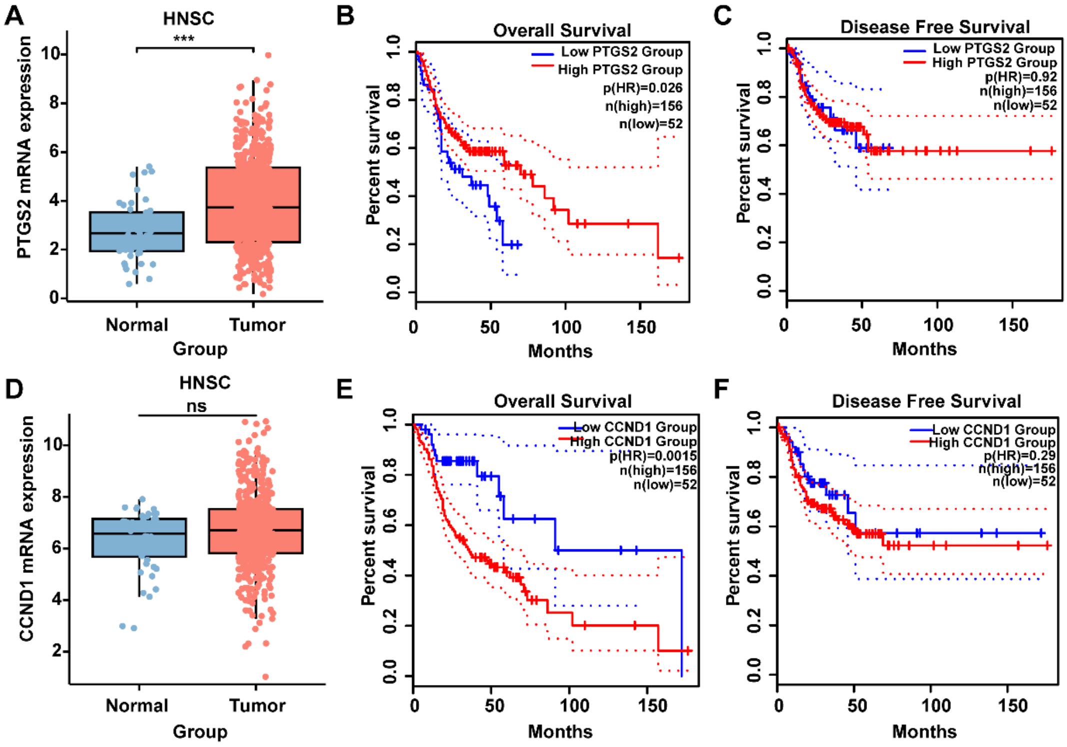

SFig 1 The upregulation of PTGS2 expression was associated with the survival rates of HNSC. **(A)** Analysis of PTGS2 expression in HNSC and its adjacent normal tissues was conducted using the TCGA database. **(B)** The relationship between PTGS2 expression and the five-year survival rate. **(C)** The association between PTGS2 expression and disease-free survival. **(D)** The expression of CCND1 in HNSC and its adjacent normal tissues was assessed using the TCGA database. **(E)** The relationship between PTGS2 expression and the five-year survival rate. **(F)** The connection between PTGS2 expression and disease-free survival. ****P* < 0.001; ns: not statistically significant.
